# Metabolic Changes on the Acquisition of Desiccation Tolerance in Seeds of the Brazilian Native Tree *Erythrina speciosa*

**DOI:** 10.3389/fpls.2019.01356

**Published:** 2019-10-23

**Authors:** Aline F. Hell, Fernanda S. Kretzschmar, Kelly Simões, Arnd G. Heyer, Claudio J. Barbedo, Marcia R. Braga, Danilo C. Centeno

**Affiliations:** ^1^Curso de Pós-Graduação em Biodiversidade e Meio Ambiente do Instituto de Botânica de São Paulo, São Paulo, Brazil; ^2^Centro de Ciências Naturais e Humanas, Universidade Federal do ABC, São Bernardo do Campo, Brazil; ^3^Programa de Pós-Graduação em Biologia Celular e Estrutural, Universidade Estadual de Campinas (Unicamp), Campinas, Brazil; ^4^BASF S.A., Santo Antônio de Posse, Brazil; ^5^Department of Plant Biotechnology, Universität Stuttgart, Stuttgart, Germany; ^6^Núcleo de Pesquisa em Sementes, Instituto de Botânica, São Paulo, Brazil; ^7^Núcleo de Pesquisa em Fisiologia e Bioquímica, Instituto de Botânica, São Paulo, Brazil

**Keywords:** carbohydrates, cell wall, legume, metabolic profiling, non-aqueous fractionation, seed maturation

## Abstract

Erythrina speciosa Andrews (Fabaceae) is a native tree of Atlantic forest from Southern and Southeastern Brazil. Although this species is found in flooded areas, it produces highly desiccation tolerant seeds. Here, we investigated the physiological and metabolic events occurring during seed maturation of E. speciosa aiming to better understand of its desiccation tolerance acquisition. Seeds were separated into six stages of maturation by the pigmentation of the seed coat. Water potential (WP) and water content (WC) decreased gradually from the first stage to the last stage of maturation (VI), in which seeds reached the highest accumulation of dry mass and seed coat acquired water impermeability. At stage III (71% WC), although seeds were intolerant to desiccation, they were able to germinate (about 15%). Desiccation tolerance was first observed at stage IV (67% WC), in which 40% of seeds were tolerant. At stage V (24% WC), all seeds were tolerant to desiccation and at stage VI all seeds germinated. Increased deposition of the arabinose-containing polysaccharides, which are known as cell wall plasticizers polymers, was observed up to stage IV of seed maturation. Raffinose and stachyose gradually increased in axes and cotyledons with greater increment in the fourth stage. Metabolic profile analysis showed that levels of sugars, organic, and amino acids decrease drastically in embryonic axes, in agreement with lower respiratory rates during maturation. Moreover, a non-aqueous fractionation revealed a change on the proportions of sugar accumulation among cytosol, plastid, and vacuoles between the active metabolism (stage I) and the dormant seeds (stage VI). The results indicate that the physiological maturity of the seeds of E. speciosa is reached at stage V and that the accumulation of raffinose can be a result of the change in the use of carbon, reducing metabolic activity during maturation. This work confirms that raffinose is involved in desiccation tolerance in seeds of E. speciosa, especially considering the different subcellular compartments and suggests even that the acquisition of desiccation tolerance in this species occurs in stages prior to the major changes in WC.

## Introduction

Physiological deterioration of seeds stored in gene banks is a huge problem that remains to be solved for large-scale, long-term seed preservation (Chmielarz, 2009). Seeds and fruits that do not mature and the difficulty to determine maturity are included among the biological constraints of reforestation, because of the requirement of a constant supply of high-quality seeds (Owens, 1995). The degree of seed orthodoxy or recalcitrance depends on the maturation degree at shedding and the characteristics of each species (Barbedo, 2018). Therefore, the understanding physiological behavior of seeds regarding desiccation tolerance is crucial to obtain seeds of high quality.

Desiccation is a natural process that occurs at the end of seed development preparing it to enter in a quiescent stage (Dasgupta et al., 1982; Beardmore and Whittle, 2005). Developing orthodox seeds acquire the ability to tolerate desiccation during the mid to late maturation stages, and their longevity increases logarithmically with the decrease in water content, allowing these seeds to retain vigor and viability throughout a storage period under defined conditions (Berjak and Pammenter, 1999; Berjak and Pammenter, 2008). However, acquisition of desiccation tolerance seems to be strongly influenced by environmental conditions and variable among different stages of development (Huang et al., 2012; Leduc et al., 2012; Barbedo et al., 2013; Caccere et al., 2013; Lamarca et al., 2013; Newton et al., 2013).

Seed desiccation is a very active event concerning gene expression and metabolism (Angelovici et al., 2010). The overlap of the metabolic processes associated with the desiccation tolerance and the events related to seed development represents the main hindrance in the study of the factors implicated in desiccation tolerance acquisition (Farrant et al., 2012). Metabolic changes occur during seed maturation, prior to dehydration, in a process dedicated to reserve synthesis and coupled to nutrient absorption (Borisjuk et al., 2004). This step of the development is accompanied by sucrose accumulation, cell expansion, and increase in the energetic status and metabolic fluxes in direction to storage products (Weber et al., 2005). At the metabolic levels, the reserve accumulation stage is characterized by a low oxygen state that causes a transitory stimulus for fermentative metabolism (Rolletschek et al., 2004). In *Arabidopsis* seeds, accumulation of storage compounds and decrease in sugars, organic acids, and amino acids were observed during the maturation stage, although alterations in these compounds were also found during seed desiccation (Fait et al., 2006). In pea (Rogerson and Matthews, 1977) and beans (Farrant et al., 1997), a decrease in the levels of sugars, followed by a decrease in respiratory rates, was observed during seed maturation. In *Arabidopsis*, a decrease in sugars and in respiratory rates, indicated by a decrease in intermediates of the Krebs cycle, was also observed (Fait et al., 2006).

Desiccation tolerance is acquired while reserve deposition occurs prior to the onset of maturation drying. Several cellular and metabolic processes have been connected with the acquisition of desiccation tolerance. They include changes in intracellular characteristics, such as reduction of the vacuolation degree, intracellular de-differentiation, decay of metabolic activity, presence of oleosine, and free-radical scavengers, as well as repair mechanisms during rehydration (Berjak and Pammenter, 1999; Oliver et al., 2000). Other important compounds, such as late embryogenesis abundant (LEA) proteins (Kermode, 1997; Wolkers et al., 2001), amphipathic molecules (Hoekstra et al., 1997), and soluble sugars and cyclitols (Koster and Leopold, 1988; Crowe et al., 1992; Centeno et al., 2016), are also considered important protective substances, mainly at the dehydrated state. Recently, a number of transcriptomic and proteomic studies have confirmed the abundance of such protective compounds associated with acquisition of desiccation tolerance in orthodox seeds (reviewed by Giarola et al., 2017).

The accumulation of sucrose and oligosaccharides during seed development, for instance, has been associated with desiccation tolerance in various species, i.e. *Brassica campestris* (Leprince et al., 1990), maize (Chen and Burris, 1990; Brenac et al., 1997), wheat (Black et al., 1999), lupin (Górecki et al., 1997), and pea (Corbineau et al., 2000). During seed drying, the formation of a glassy matrix might reduce the molecular mobility as an aspect on the protection of lipid membranes and macromolecules (Williams and Leopold, 1989; Buitink et al., 1999). Soluble sugars, such as sucrose and oligosaccharides, may form a glass during desiccation, resulting in molecular stabilization during desiccation (Williams and Leopold, 1989; Wolkers et al., 1998). This could even determine the longevity of the seed since this would vary according to the newly formed spatial interactions among molecules during desiccation (Walters, 2015). Moreover, vitrification of the intermembrane sugar solution could improve the resistance of the lipid phase transition in membranes during dehydration process (Bryant et al., 2001). In leaves of *Arabidopsis*, a non-aqueous fractionation (NAF) showed that raffinose is accumulated in the plastids after freeze–thaw cycles, and its role might be related to the stabilization of the photosystem II, located at plastid membranes (Knaupp et al., 2011). Cold acclimation also resulted in the accumulation of raffinose and galactinol in plastids fractions (Hoermiller et al., 2017). The accumulation of both compounds has been also related to work against deleterious effects of reactive oxygen species (ROS) in *Arabidopsis* (Nishizawa et al., 2008). In contrast, monosaccharide levels have a negative correlation with desiccation tolerance (Hoekstra et al., 2001). Fructose and glucose could be involved in Maillard reactions and would be a source of hydroxyl radicals in dehydrated tissues, thus favoring the appearance of browning (Koster and Leopold, 1988; Van der Toorn and McKersie, 1995). In *Medicago truncatula*, Vandecasteele et al. (2011) have suggested that the increase in sucrose/raffinose family oligosaccharides (RFO) ratio can negatively affect seed vigor.

Cell wall folding has been considered as another strategy to overcome cell desiccation, preventing possible rupture of the continuum cell wall-plasma membrane during water loss and after rehydration (Webb and Arnott, 1982). The extent and way of folding in related to their conformation and chemical composition. High proportions of arabinans, which are highly mobile polysaccharides and are able to absorb water, allow for higher wall flexibility, thus contributing to diminish mechanical stress during water loss (Moore et al., 2006; Farrant et al., 2012). Increased deposition of arabinose-containing polymers has been reported during development of orthodox seeds, e.g., of *Arabidopsis* (Gomez et al., 2009) and *Tyslosema esculentum* (Mosele et al., 2011), suggesting that they might play a role in seed tissues that undergo desiccation.

Although many studies with seeds, carried out during the last several years, have contributed to a better understanding of some events connected to desiccation tolerance acquisition, most information has been gathered from experiments performed with crops. Seeds of tropical species show differences in the degree of desiccation tolerance at shedding. Frequently, these differences occur among seeds of the same species from different regions and even from different years from the same specimen (Daws et al., 2004; Lamarca et al., 2013). Tropical forests constitute a genetic diversity reservoir, which plays a crucial role in the environment stability. Most seeds are disseminated with high water content, and germination often occurs directly after dissemination, although seeds with hard coats, such as many legume and wind dispersed seeds from dry fruits, show orthodox storage behavior (Rodríguez et al., 2000).

*Erythrina speciosa* is a legume tree native of the Brazilian Atlantic Forest with ornamental potential and rich in bioactive secondary metabolites, which have been used in traditional medicine due to their antimicrobial, antimalarial, and antiparasitic properties (Amaral et al., 2019). Although this species is found in flooded areas, its seeds are remarkably tolerant to desiccation and can be stored for many years under different conditions without losing vigor. These orthodox seeds have dormancy by seed coat water impermeability, which varies according to the climate conditions during maturation (Molizane et al., 2018). The mature seeds of *E. speciosa* are composed of ca. 40% soluble carbohydrates, which are mainly represented by raffinose family oligosaccharides (45% of raffinose, 40% of sucrose 12% of stachyose) and small amounts of reducing sugars, such as glucose and fructose. Starch represents less than 2% of the storage material and lipids account for ca. 12% (Mello et al., 2010; Mello et al., 2011). A number of studies performed by our group with *E. speciosa* allowed us to understand the physiological characteristics and the biochemical composition of its seeds during maturation and after germination (Mello et al., 2010; Mello et al., 2011; Molizane et al., 2018). Thus, *E. speciosa* has become a potential interesting model for studies of classical orthodox seeds from tropical environments that accumulate mainly soluble sugars. The lack of reports of the metabolic changes associated to water loss in seeds of this species prompted us to investigate the physiological and metabolic events occurring during seed maturation in E. speciosa to establish a better understanding of the fundamental changes related to its acquisition of desiccation tolerance.

## Materials and Methods

### Plant Material and Sampling

**Figure 1:**
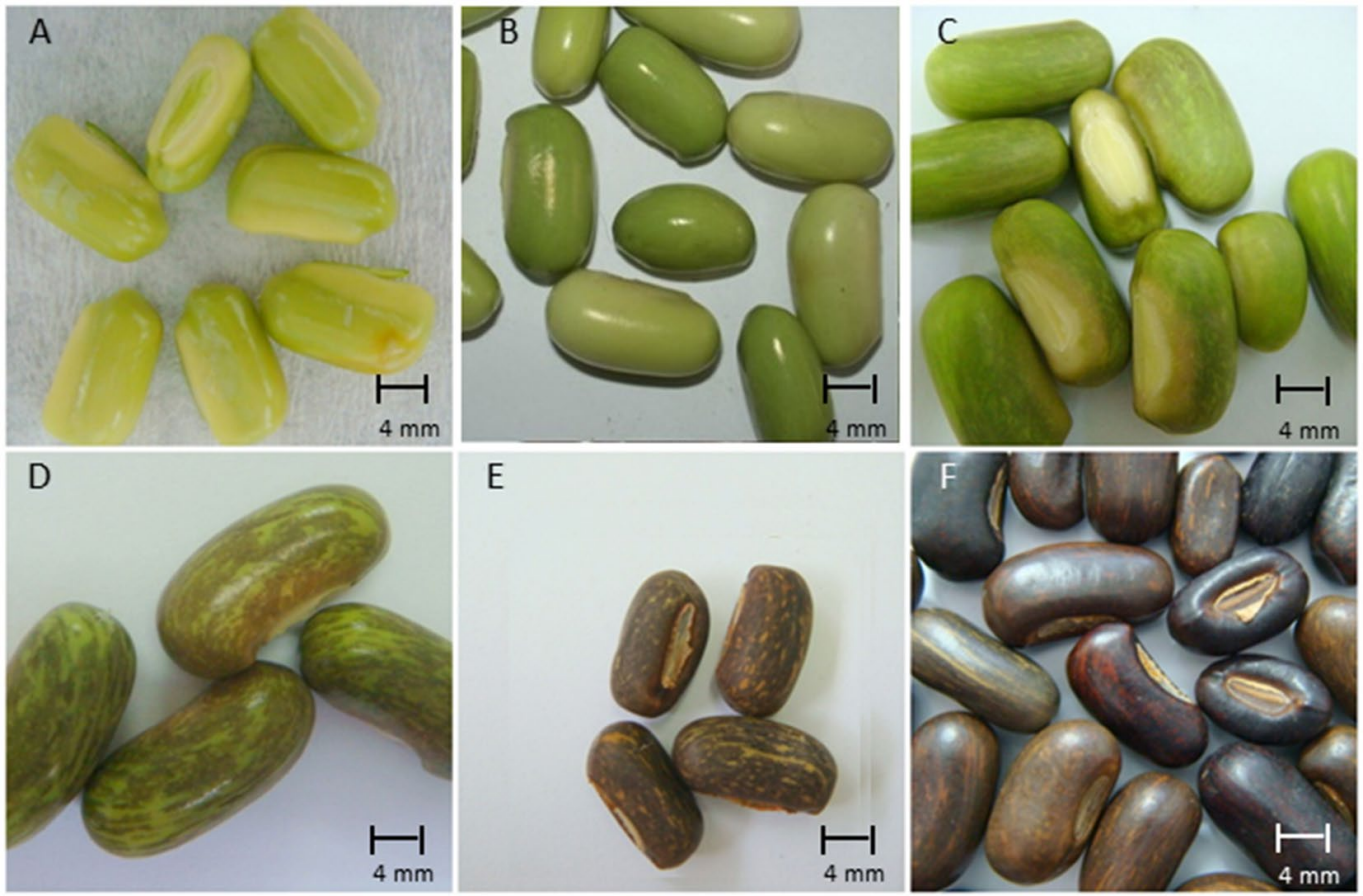
Morphological aspects of seeds of *Erythrina speciosa* at six maturation stages (**A**: 20 DAA, stage I; **B**: 38 DAA, stage II; **C**: 42 DAA, stage III; **D**: 44 DAA, stage IV; **E**: 57 DAA, stage V; **F**: 60 DAA, stage VI) obtained in a single harvest.

Fruits of *E. speciosa* Andrews were collected from fully grown plants growing in a homogeneous plantation located at the Parque Central de Santo André (23°40′ 20–50′′ S and 46°31′35–55′′W, 784 m alt.), state of São Paulo, Brazil, during the whole fruiting season, which lasts from August to October, the end of winter and beginning of spring in the Southern hemisphere. In the first year experiment, flowers were tagged on the day of their anthesis (DAA), and the development of fruits and seeds was analyzed by collecting fruits directly from the branches and classified as 20, 38, 42, 44, 57, and 60 DAA. Seeds were removed from the pods by hand, and their length, width, thickness, and color were also registered (four replicates of 10 seeds) before the evaluation of their morphological characterization (**Supplementary Figure S1**). Each sampling period was considered as a different stage of the seed development. In the second year experiment, based on the results obtained in the first experiment, six different stages (I to VI) of seed development were obtained from a single harvest. These six stages cover the end of the embryogenesis and the entire maturation process (**Figure 1**).

### Analysis of Seed Desiccation Tolerance

Seeds from each stage of development were submitted to drying up to about 15% and 10% water content by closing them in desiccators with dry silica gel, at 25°C. After reaching the target water content, germination testes were carried out as described previously.

### Seed Maturation and Germination

Seeds were weighed, and the water content (%, on a fresh weight basis) and the dry matter (mg seed^-1^) were determined for each stage of maturity (four replicates of four seeds), after oven drying at 103°C ± 3°C for 24 h (ISTA, 2015). Before drying, the water potential of seeds was measured with a Decagon WP4 potentiometer (Pullman, USA) based on the dew point (Decagon Devices 2001), according to the manufacturer’s instructions. For this procedure, lengthwise sectioned seeds were measured, using four replicates of five seeds each (Mello et al., 2011). Seeds from six different stages of development were submitted to germination tests in paper rolls. Four replicates of 15 seeds were placed between two layers of germination paper previously moistened with distilled water. After rolling the papers, the rolls were maintained in Marconi MA400 germination chambers (Piracicaba, Brazil) at 25°C ± 1°C, under continuous light. Germination was evaluated every 2 days from the sowing day, by registering the protrusion of the primary root (≥5 mm). After 30 days, the normal seedlings development was registered considering seeds with both normal radicular (single straight primary root) and shoots system (normal leaf morphology for the species). To estimate vigor, the Germination Speed Index (GSI) was calculated according Maguire’s equation (Maguire, 1962), GSI = n1 ⁄t1 + n2 ⁄ t2 + … n7 ⁄ t7; where n1, n2, … n7 are the number of germinated seeds at times t1, t2, … t7 (in days).

### Sugar Extraction and Analysis

Seeds from the six different stages of development had their seed coat removed before the embryos were separated in embryonic axis and cotyledon and weighed for subsequent analyses. Embryonic axes and cotyledons from 30 fresh embryos (in triplicates) were boiled separately in 80% ethanol (3 ml·g^−1^·fresh mass^−1^) for 5 min for enzyme inactivation. The supernatants were recovered by centrifugation (1000*g* for 20 min), and the residues were manually homogenized and re-extracted twice in boiling 80% ethanol. After centrifugation, the residues were washed with distilled water and recovered by centrifugation. The resulting ethanolic and aqueous supernatants were combined, concentrated in rotoevaporator, re-dissolved in 10 ml of distilled water and considered as the soluble sugar extracts. Residues were freeze-dried and used for quantification of starch and cell wall fractionation as described below. The amounts of total carbohydrates and reducing sugars in the ethanolic extracts were determined colorimetrically by the phenol-sulfuric acid method (Dubois et al., 1956) and Somogyi-Nelson procedure (Somogyi, 1945), respectively, using glucose as standard.

### Cell Wall Extraction and Analysis

Aliquots of the ethanol-insoluble residue were extracted three times with chloroform: methanol (1:1, v/v) (10 ml·g^−1^) at room temperature for 20 min, followed by extraction with acetone and ethyl ether for 10 min to remove lipids.

The residue after lipid removal was extracted with 90% dimethyl sulfoxide (DMSO) (10 ml·g^−1^·dry residue^−1^) for 16 h at room temperature. After centrifugation at 13,000*g* for 15 min, the residue was submitted to four additional extractions with DMSO, twice at room temperature for 4 h and twice overnight at 5°C. The residue was then exhaustively washed with distilled water, centrifuged, and subsequently treated with amyloglucosidase from *Aspergillus niger* (Megazyme, Ireland) in sodium acetate buffer 100 mM, pH 4.5 (0.1 unit enzyme mg^-1^) for 24 h at 30°C to remove the remaining starch after the DMSO extraction. The residue was washed thoroughly with distilled water (10 ml·g^−1^), chloroform: methanol (1:1, v/v), and acetone followed by ethyl ether (twice), acetone, and was recovered by filtration through a glass fiber filter (Whatman GF/A). Finally, the residue was freeze-dried and considered as the yield in crude cell walls (modified from Gorshkova et al., 1996).

One milligram of crude cell walls from embryonic axis and cotyledons was hydrolyzed in 500 µl 2 N trifluoroacetic acid (TFA) in an autoclave at 121°C (1.5 atm, 1 h). The acid was eliminated by evaporation, and the residue was dissolved in deionized water for further anion exchange chromatography analysis. The amounts of uronic acids were determined colorimetrically by *m*-hydroxydiphenyl assay (Filisetti-Cozzi and Carpita, 1991) using galacturonic acid (Sigma Co., USA) as standard.

Neutral sugars were analyzed by anion exchange chromatography in an ICS 3000 system (Dionex, USA), coupled with a pulsed amperometric detector (HPAEC/PAD) (Dionex, USA). The neutral monosaccharides were eluted isocratically in a Carbo-Pac PA1 column in 20 mM NaOH at a flow rate of 0.2 ml·min^−1^, for 35 min and identified by comparing their elution times with commercial standards of fucose, arabinose, rhamnose, galactose, glucose, xylose, and mannose (Sigma Co., USA).

### Metabolic Profile

The metabolic profiling was performed by GC-MS as described by Roessner et al. (2001), modified with the peak identification optimized for *E. speciosa* seeds. Due to the very low mass of embryonic axes from stage I, the metabolic profile was obtained for stage II to VI. Cotyledons and embryonic axes separated from seeds were ground in liquid nitrogen, 100 and 50 mg were weighed, respectively, and extracted in 500 µl of chloroform/methanol/water mix (12:5:1) and 50 µl of adonitol (0.2 mg·ml^−1^ pyridin) as internal standard. The mixture was agitated in a vortex, warmed at 60°C for 30 min under agitation and centrifuged at 13,000 rpm for 2 min. From the upper phase (hydroalcoholic), 350 µl were then transferred to a new tube, and 350 µl of water were added. The mixture was then agitated and centrifuged at 13,000 rpm for 5 min. Three hundred microliters were taken and dried under vacuum for further derivatization. The dried samples were derivatized with 150 µl of pyridin, 50 µl of N,O-Bis(trimethylsilyl)trifluoroacetamide (BSTFA), and 50 µl of methoxiamine hydrochloride (0.2 mg·ml^−1^ pyridin) and injected in a gas chromatography–mass spectrometry (GC-MS) system composed of an Agilent GC 6890 series (Agilent, USA). GC was performed on a 30-m HP5 column with 0.25 µm film thickness (Supelco, Bellfonte, CA). The injection temperature was set at 230°C, the interface at 250°C, and the ion source adjusted to 150°C. Helium was used as the carrier gas at a flow rate of 1 ml·min^−1^. The analysis was performed under the following temperature program: 5 min of isothermal heating at 70°C, followed by a 5°C·min^−1^ oven temperature ramp to 310°C, and a final 1 min of heating at 310°C. Mass spectra were recorded at 2 scan s^-1^ with a scanning range of 50 to 600 m/z. Both chromatograms and mass spectra were evaluated using the ChemStation program (Agilent, USA). The peaks were identified and quantified in comparison with authentic standards and the NIST Mass Spectral Library.

### Estimation of Respiratory Rates Analysis

Respiratory rates were estimated using approximately 90 seeds per developmental stage, separated in three replicates. The O_2_ consumption and CO_2_ release were determined by an analyser model 6600 (Illinois Instruments, Inc., Johnsburg, EUA) as previously described by Lamarca and Barbedo (2012).

### NAF

Subcellular fractionation of vacuolar, plastidial, and cytosolic compartments was done according to Knaupp et al. (2011). Briefly, 80 mg of freeze-dried tissue homogenate was suspended in 10 ml of heptane-tetrachlorethylene (ρ = 1.3 g cm^3^), cooled on ice, and repeatedly sonified for 5 s with pauses of 15 s over a time course of 12 min (Branson Sonifier 250, output control 4; Branson). Subsequently, the sonified suspension was passed through nylon gauze, pore size 30 μm, and centrifuged. The sediment was re-suspended in heptane-tetrachlorethylene (ρ = 1.3 g cm^3^) and loaded on a linear gradient of heptane-tetrachlorethylene (ρ = 1.3 g cm^3^) to tetrachlorethylene (ρ = 1.6 g cm^3^). After ultracentrifugation at 121,000*g* for 3 h, the gradient was fractionated into nine 1-ml fractions. These were divided into three subfractions of 0.3 ml and dried under vacuum. One subfraction was used for marker enzyme determination and another one for metabolite analysis. Alkaline pyrophosphatase as plastidial marker, UGPase as a cytosolic marker, and acid phosphatase as marker for the vacuolar compartment were measured essentially as described (Knaupp et al., 2011).

### Statistical Analysis

The results were analyzed by applying *F* test (*p* < 0.05 as significance threshold) in an entirely randomized design, with four replicates, and a Tukey test (5%) was applied among treatments. For NAF of seed subcellular compartments, a correlation analysis of marker enzyme activities and metabolite distributions using Kendall’s rank correlation (Kendall, 1938) was performed, applying a significance threshold of p < 0.01.

## Results

**Figure 2:**
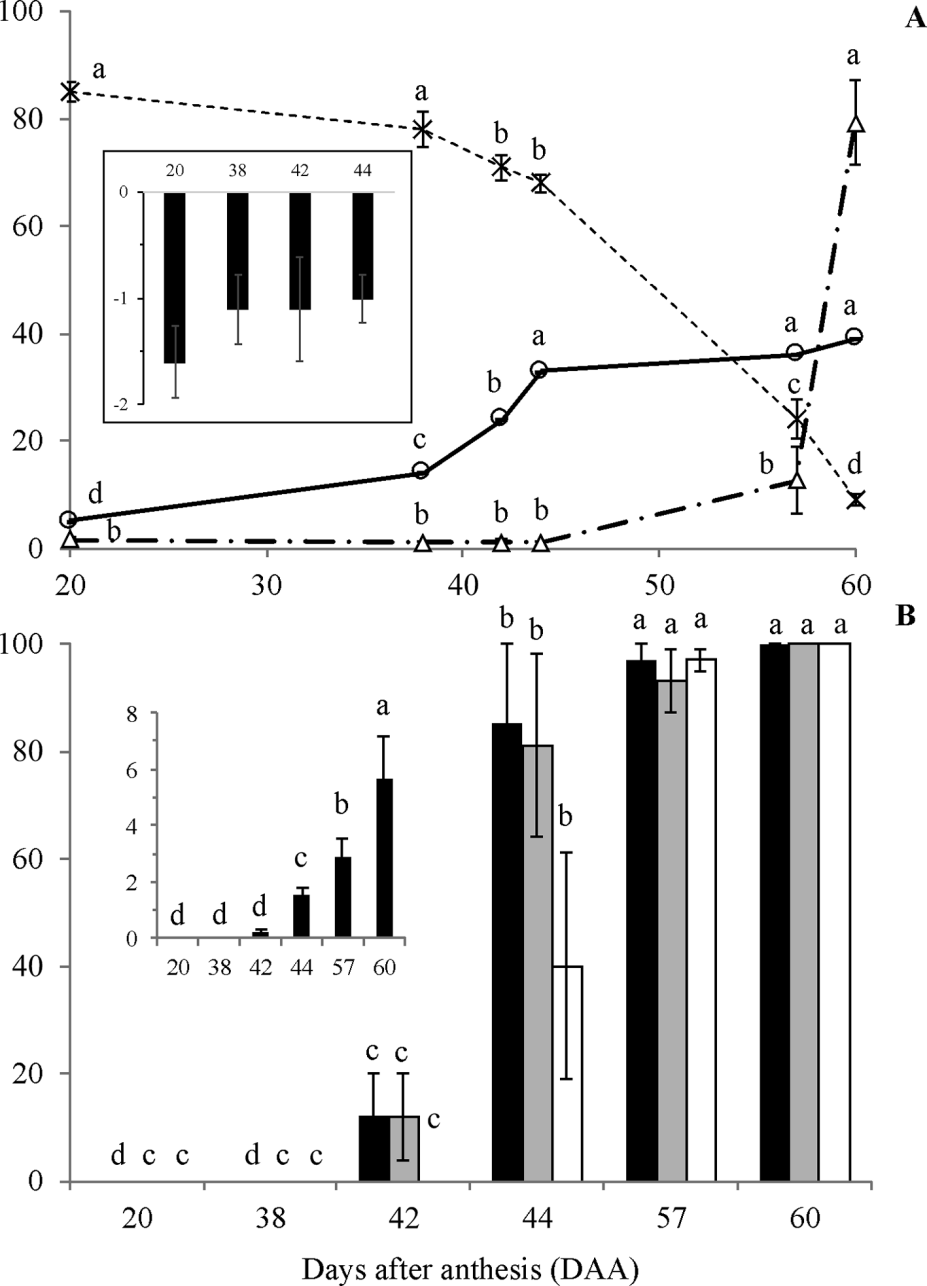
Water content (**A**, –x–, %), dry mass (**A**, –o–, g/100 seeds), water potential (**A**, Δ, -MPa; A, small chart), germination (**B**, black columns, %), normal seedling development (**B**, grey columns, %), germination of dried seeds (**B**, white columns, %) and Germination Speed Index (**B**, small chart) of *Erythrina speciosa* obtained from stages I to VI. Four biological replicates (containing at least four seeds each) were used for water content, dry mass and water potential, and four biological replicates (containing 15 seeds each) were used for germination and normal seedling development analysis. Different letters indicate statistical difference among the developmental stages by Tukey test (5%).

Although some increase in length and diameter was observed in fruits of *E. speciosa* from 20 to 30 DAA and from 20 to 45 DAA, respectively, fruits had almost achieved the maximum size already at the 20 DAA (**Supplementary Figure S1**). Conversely, seeds increased size substantially till 45 DAA, mainly because of the gain in dry matter (**Figure 2A**). From 45 DAA on, the seed size declined until 55 DAA (**Supplementary Figure S1**), mainly due to reduction in water content (**Figure 2A**). Indeed, water content is stable until 38 DAA, when an almost linear drop until the last stage (60 DAA) was observed (**Figure 2A**). Water potential remained constant until 44 DAA (**Figure 2A**) and decreased after this time point, reaching the lowest value at the end of seed development. The changes in water potential were not high as those observed for water content from 44 to 57 DAA. Neither the water content changed as much as water potential from 57 to 60 DAA. Thus, it seems that water status was not only dependent on water content but other active processes seemed to occur to maintain water flux from mother plant to the seeds. The results also showed that an important period to be considered in *E. speciosa* seed maturation is the interval from 40 to 55 DAA, when most changes occur.

The physiological behavior of *E. speciosa* seeds during maturation followed the classical pattern described for orthodox seeds, i.e., decrease of water content from more than 80% to *ca.* 10% at the end of maturation (**Figure 2A**). The dry mass accumulation was the same as expected for orthodox seeds, increasing from 20 to around 44 DAA (**Figure 2A**).

Seeds became germinable and produced normal seedlings around 42 DAA (**Figure 2B**), with increasing germination rates until 57 DAA. However, maximum vigor was reached only at the latest stage, 60 DAA (**Figure 2B**). Also, the results of germination after seed drying showed that desiccation tolerance started about 44 DAA, where 40% of the seeds were tolerant (**Figure 2B**). At 57 DAA, all seeds were able to germinate after drying. Despite showing about 15% of germination, 42 DAA seeds did not tolerate drying.

In the embryonic axis, there was a remarkable increase in the cell wall yield during seed maturation from stage II to stage IV (**Table 1**). Cell wall yield represents the amount of dry mass that is present as cell wall, and the proportion on the overall seed dry mass was 3.4 times lower in stage II compared with stage VI. In contrast, a decrease in the cell wall yield was observed in the cotyledons during seed maturation. The proportion of cell wall was ca. 1.4 lower in stage VI in comparison with stage I. The content of uronic acids decreased in the cell walls of both, embryonic axis and cotyledons, subsequent to stages II and I, respectively.

**Table 1:** Cell wall yield, uronic acid content, and relative percentage of neutral monosaccharides in embryonic axis and cotyledons of *Erythrina speciosa* seeds during different stages (I-VI) days after anthesis (DAA).

DAA	Embryonic axis						Cotyledons					
	I	II	III	IV	V	VI	I	II	III	IV	V	VI
Cell wall yield (mg g seed DM^-1^)												
	–	144.7a	355.8b	473.9c	473.9c	488.1c	388.1ab	436.6a	345.8bc	374.4ab	377.9ab	280.1c
Content of uronic acids (mg g cell wall DM^-1^)												
	–	29.7a	22.2b	20.9b	21.6b	22.9b	35.7a	19.0b	22.2ab	22.5ab	21.7b	25.4ab
Neutral sugars (relative %)												
Fucose	–	1.7a	1.1a	0.9a	1.0a	1.0a	1.0a	1.2a	1.0a	0.9a	1.3a	1.3a
Rhamnose	–	4.1a	2.6b	2.3b	2.4b	2.4b	2.3ab	2.5b	1.6a	1.7a	1.8ab	1.6ab
Arabinose	–	18.8a	25.1b	29.1c	28.5c	27.9bc	26.1a	26.7ab	25.3a	32.4bc	30.7abc	35.8c
Galactose	–	28.5a	27.9a	26.8a	34.2b	33.9b	40.4ab	31.8c	31.0c	33.2ac	43.4b	35.8ac
Glucose	–	16.6a	14.4ab	12.2b	9.8c	9.7c	13.5a	22.4b	21.4b	10.9a	3.7c	3.8c
Xylose/mannose	–	30.3a	28.9a	28.7a	24.1b	25.1b	16.7ac	15.3c	19.6ab	20.9b	19.2b	21.1ab

Letters compare each value (line) within each organ among different maturation stages by Tukey 5%.

– not analyzed due to low yield.

Glycosyl composition analysis revealed that although xylose/mannose and galactose were the main monomers in cell walls of embryonic axis at stages II and III, arabinose increased significantly from the stage III on, predominating at stage IV (**Table 1**). The relative proportion of galactose was higher in stages V and VI compared with the beginning of seed maturation and coinciding with a significant reduction in glucose and xylose/mannose. Increased proportion of arabinose was also observed in cotyledons during seed maturation, peaking at stage IV and presenting almost the same proportion of the predominant sugar galactose. As observed in the embryonic axis, glucose was also reduced in the cotyledon cell walls at the last stages of maturation.

The content of total soluble sugars presented a remarkable increase at stages V and VI, but their levels were 3.9 times higher in embryonic axis than cotyledons (**Table 2**). At stage I, soluble sugars were represented by equal amounts of the monosaccharides fructose and glucose and the disaccharide sucrose in embryonic axis. The relative proportion of sucrose reached ca. 60% at stage II, decreasing later, probably due to its consumption for synthesis of the raffinose family oligosaccharides (RFO), raffinose, and stachyose, as indicated by values observed at the late stages of development. These RFO together summed up to 45% of the soluble sugars at stage VI. In the cotyledons, a similar tendency was observed, as sucrose decrease from stage III to VI, and accumulation of RFO was observed from stage III (**Table 2** *,* **Supplementary Figure S2**).

**Table 2:** Relative percentage of total soluble sugars, monosaccharides, and oligosaccharides in embryonic axis and cotyledons of *Erythrina speciosa* seeds during different stages (I to VI, DAA).

	Embryonic axis						Cotyledons					
Stages	I	II	III	IV	V	VI	I	II	III	IV	V	VI
(DAF)	20	38	42	44	57	60	20	38	42	44	57	60
Content soluble sugar (mg g DM^-1^)	57.7a	72.1a	39.4a	65.6a	196.7b	294.8c	21.2a	14.4a	17.3a	20.0a	58.3b	75.4c
Sugars (relative %)												
Glucose	21.0Aab	17.2Ac	20.1Ab	12.4Aa	11.4Aa	14.4Aa	24.5Ab	30.0Aab	26.3Ab	19.5Ad	24.6Ab	26.5Acd
Fructose	29.3Ab	16.4ABc	28.0ABb	42.4Ba	22.6ABbc	20.5ABa	37.9Ac	26.9Aab	28.4Ab	55.2Aab	49.6ABc	34.6ABd
Sucrose	49.7BCb	61.1Cd	26.9ABb	3.1Aa	11.9Aab	20.9Aa	37.6Bc	41.2Bb	29.6Bb	0.3Ac	0.0Aa	2.4Aa
Raffinose	0.0Aa	5.3Ab	25.0Ab	35.6Aa	32.2Ac	28.6Aa	0.0Aa	1.8Ba	15.7ABab	21.2ABa	12.9ABab	20.9ABbc
Stachyose	0.0Aa	0.0Aa	0.0Aa	6.4ABa	21.9Cabc	15.7BCa	0.0Aa	0.0Aa	0.0Aa	3.8Aa	12.9Aab	15.6Aab

Letters compare means of triplicates in each organ by Tukey 5%. Capital letters compare the sugar content among stages. Lower letter compares the relative sugar content in each stage.

**Figure 3:**
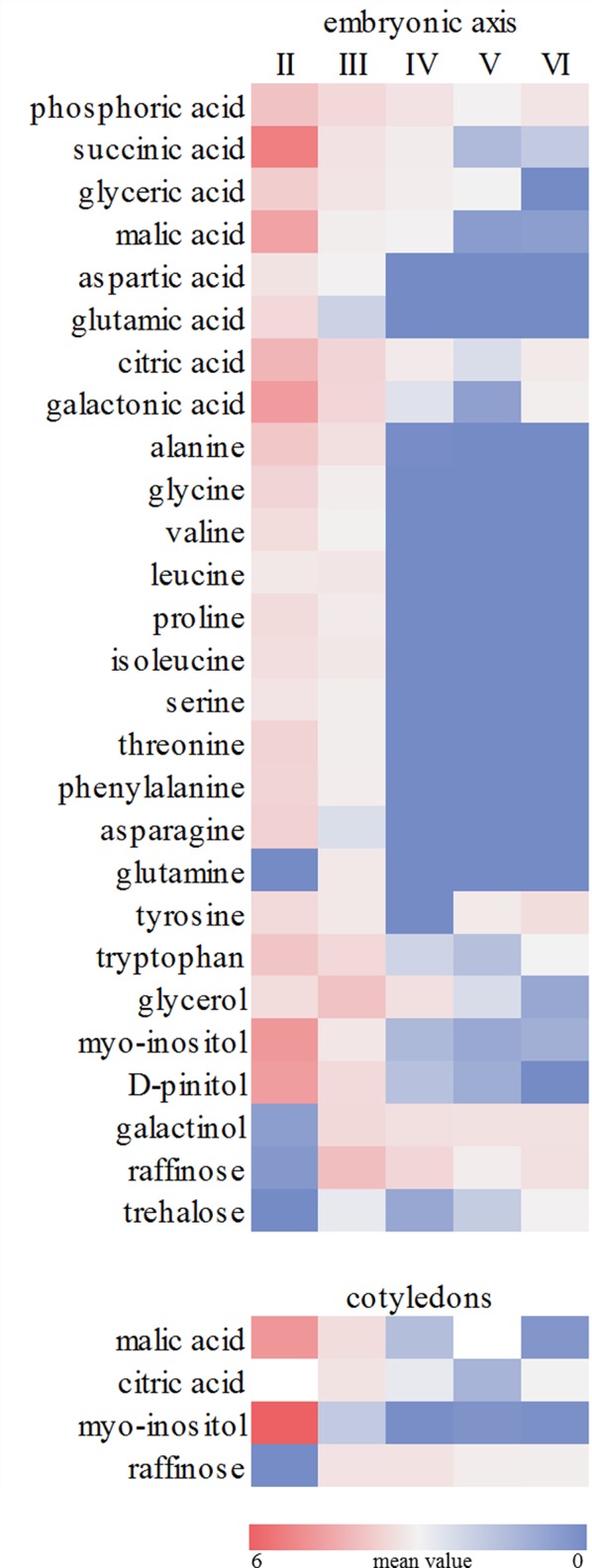
Relative abundance of metabolites. Colours represent the relative metabolite abundance among the different developmental stages (II to VI). Values are the mean of at least five (embryonic axes) or three (cotyledons) biological replicates. Red and blue colors represent values higher or lower, respectively. The scale bar indicates the magnitude of variation among the developmental stages (0- to 6-fold the mean value).

**Figure 4:**
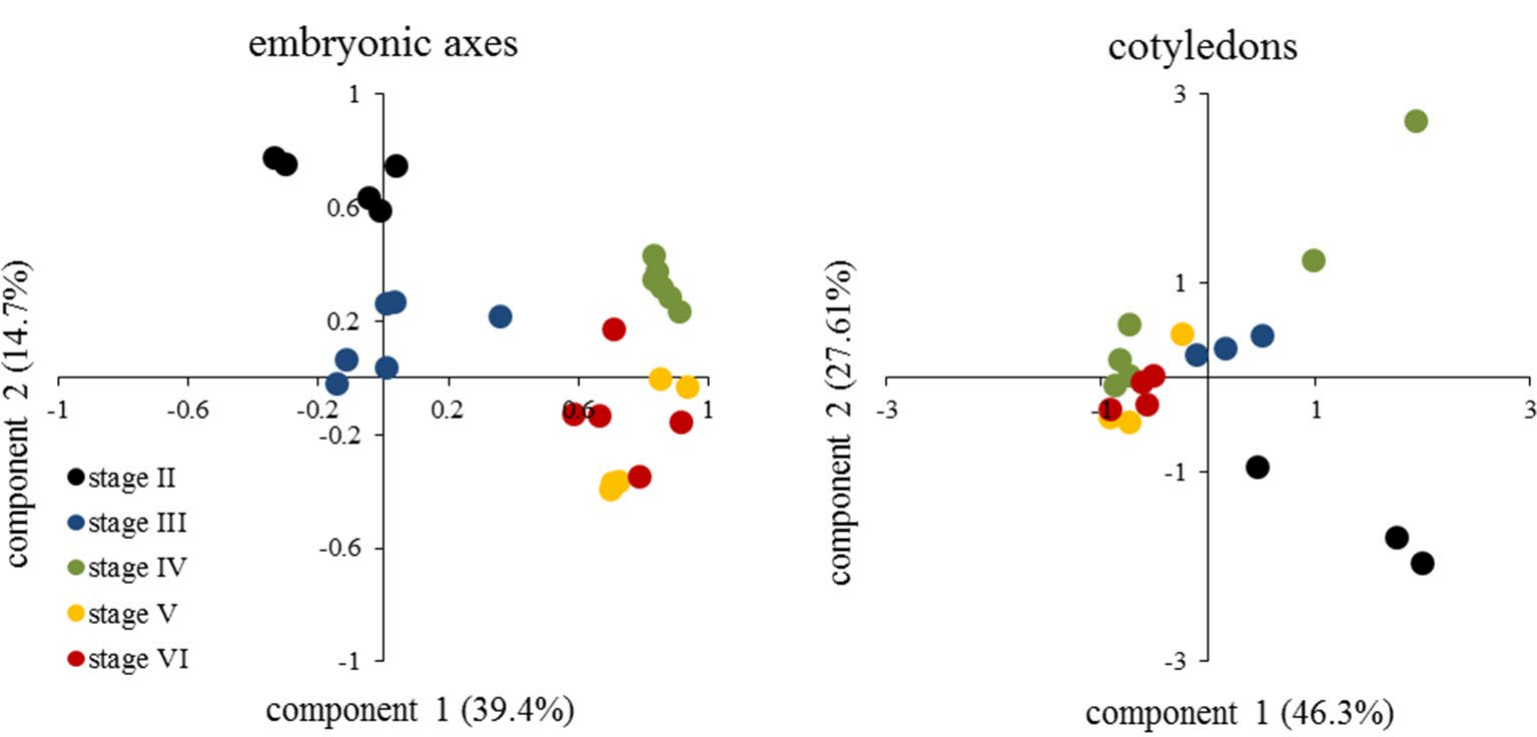
Principal Component Analysis from metabolic profile of embryonic axes and cotyledons of *Erythrina speciosa* seeds at different developmental stages (stage II to stage VI). At least five (embryonic axis) or three (cotyledons) biological replicates were used for the analysis.

**Figure 5:**
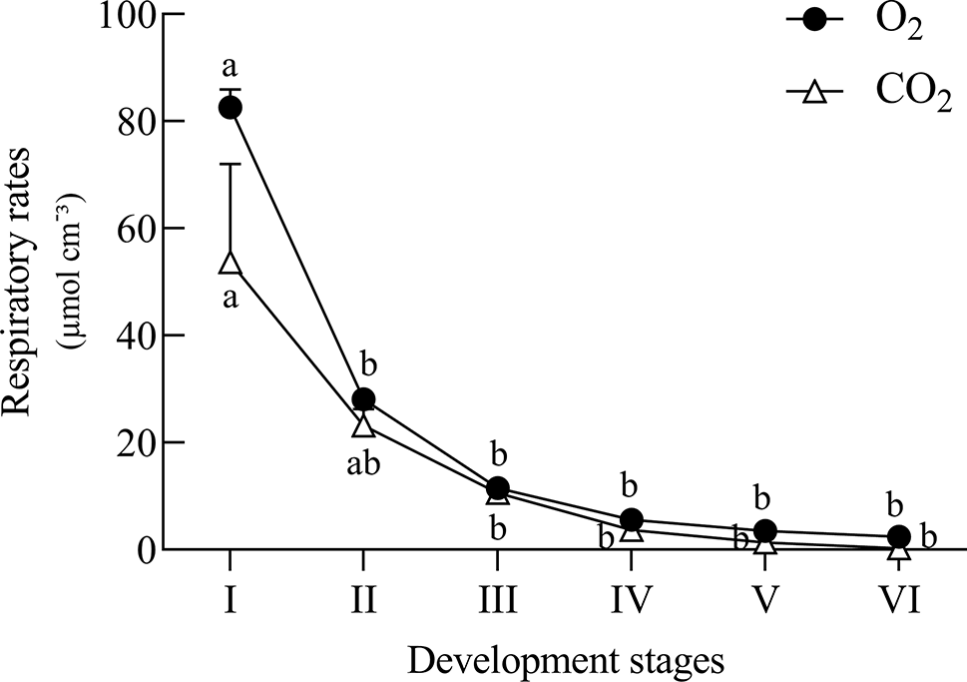
Estimated respiratory rates of *Erythrina speciosa* seeds at six developmental stages. Values are the mean of three biological replicates. Different letters indicate statistical difference among the developmental stages according to Tukey test (5%).

The metabolic profile showed a decrease in the levels of organic acids in embryonic axis of *E. speciosa* during seed development, the major changes occurred during the first stages of development (**Figure 3**). The decrease in the level of succinic, citric, and malic acids suggests a reduction in mitochondrial metabolism. Those changes were more pronounced between the stages II and III (**Figure 3**). Metabolic profiles in *E. speciosa* axis also showed a decrease in amino acids between stages II and III, from whereon these compounds were below the detection limit, except for tryptophan and tyrosine (**Figure 3**). A Principal Component Analysis (PCA), based on the relative abundance of compounds found at the metabolic profile, brings a holistic view concerning the deactivation of the metabolism (**Figure 4**). There is a great switch on the metabolism, especially on embryonic axis, from stage II to stage IV. This switch corresponds to a metabolic shutdown marked by decreased levels of TCA cycle and other primary metabolites (**Figure 3**) and is in agreement with recordings of respiratory rates (**Figure 5**).

The disaccharide trehalose was also detected only in *E. speciosa* axes, and an increase in its level was found especially between maturation stages IV and VI (**Figure 3**). Sucrose was found during all stages of *E. speciosa* seed development in both axes and cotyledons (**Figure 3**). The metabolic profiles show an antagonistic behavior of sucrose and raffinose between maturation stages II and III (**Figure 3**).

**Figure 6:**
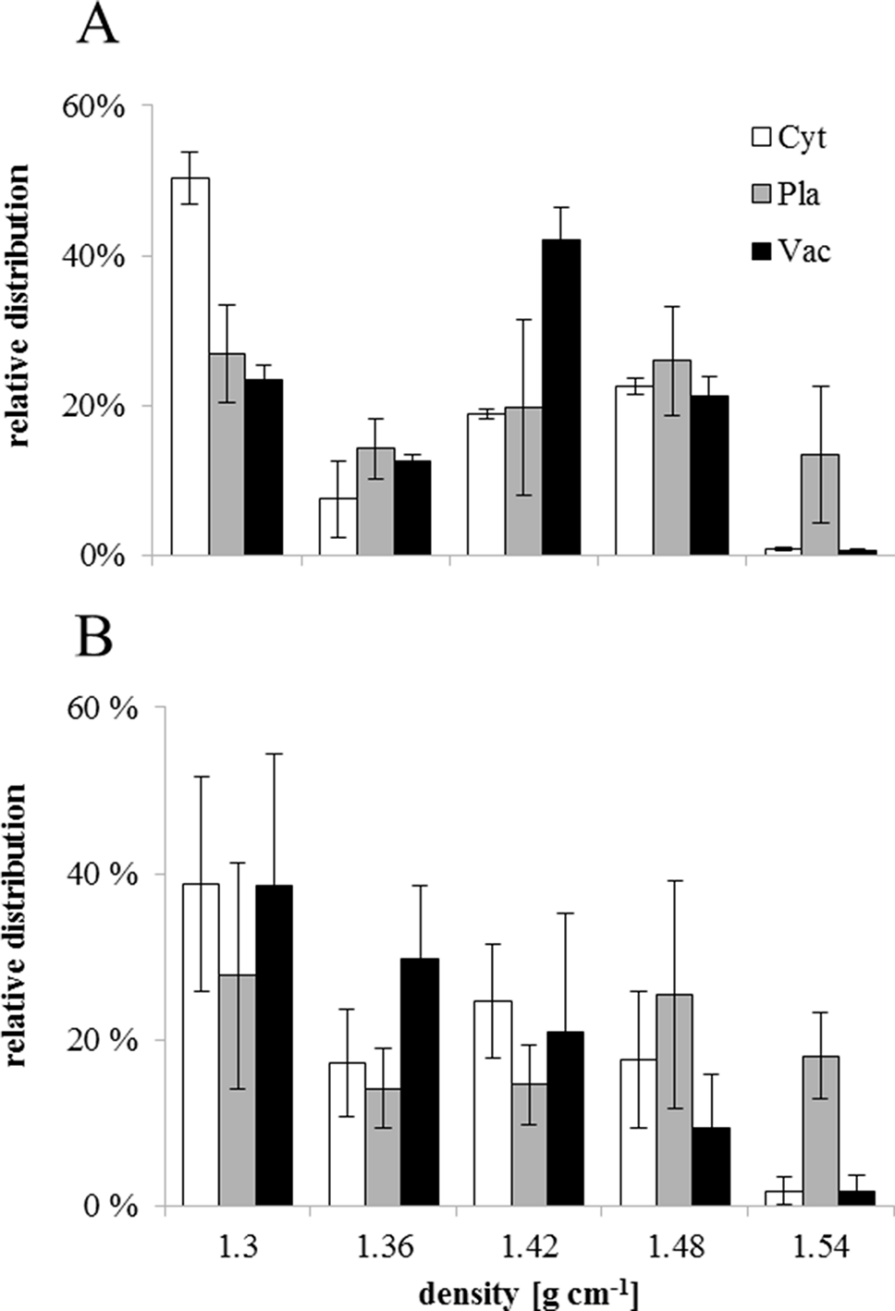
Relative distribution of sub-cellular compartments on different densities obtained by non-aqueous fractionation of *Erythrina speciosa* seeds on stage I **(A)** and stage VI **(B)**. Cyt, cytosol (white); Pla, plastids (gray); Vac, vacuole (black). Values are given as mean of the percentage ratio of four biological replicates ± standard deviation.

NAF of seed subcellular compartments based on density was performed for stages I and VI. Marker enzyme activities for plastids (alkaline pyrophosphatase), cytosol (UGPase), and vacuole (acid phosphatase) identified the cytosol as lightest compartment, while plastids showed a broad distribution. The vacuole peaked at an intermediate density of 1.42 g·ml^−1^ in stage I, while it was much lighter in stage VI, showing a distribution similar to the cytosol (**Figures 6A, B**). To prevent metabolite diffusion during compartment separation, NAF uses density gradient centrifugation of subcellular particles generated by ultra-sonication of lyophilized tissue (see Materials & Methods). This does not yield purified organelles, but a distribution over the gradient fractions, which, based on marker enzyme activities, is used for mathematical assignment of metabolites to the different compartments. Only sugars and sugar alcohols could be significantly correlated with marker enzyme activities (**Supplementary Table 1**). During stage I, hexoses, raffinose, stachyose, and *myo*-inositol showed strongest correlation with the vacuole, while raffinose was significantly associated also with the cytosol. At stage VI, however, sucrose, raffinose, and stachyose were significantly associated with cytosol and vacuole, while the sugar alcohol xylitol was found only in the vacuole. The weaker association of sugars with plastids during stage I was completely lost in stage VI (**Figures 6A, B** and**Supplementary Table 1**).

**Figure 7:**
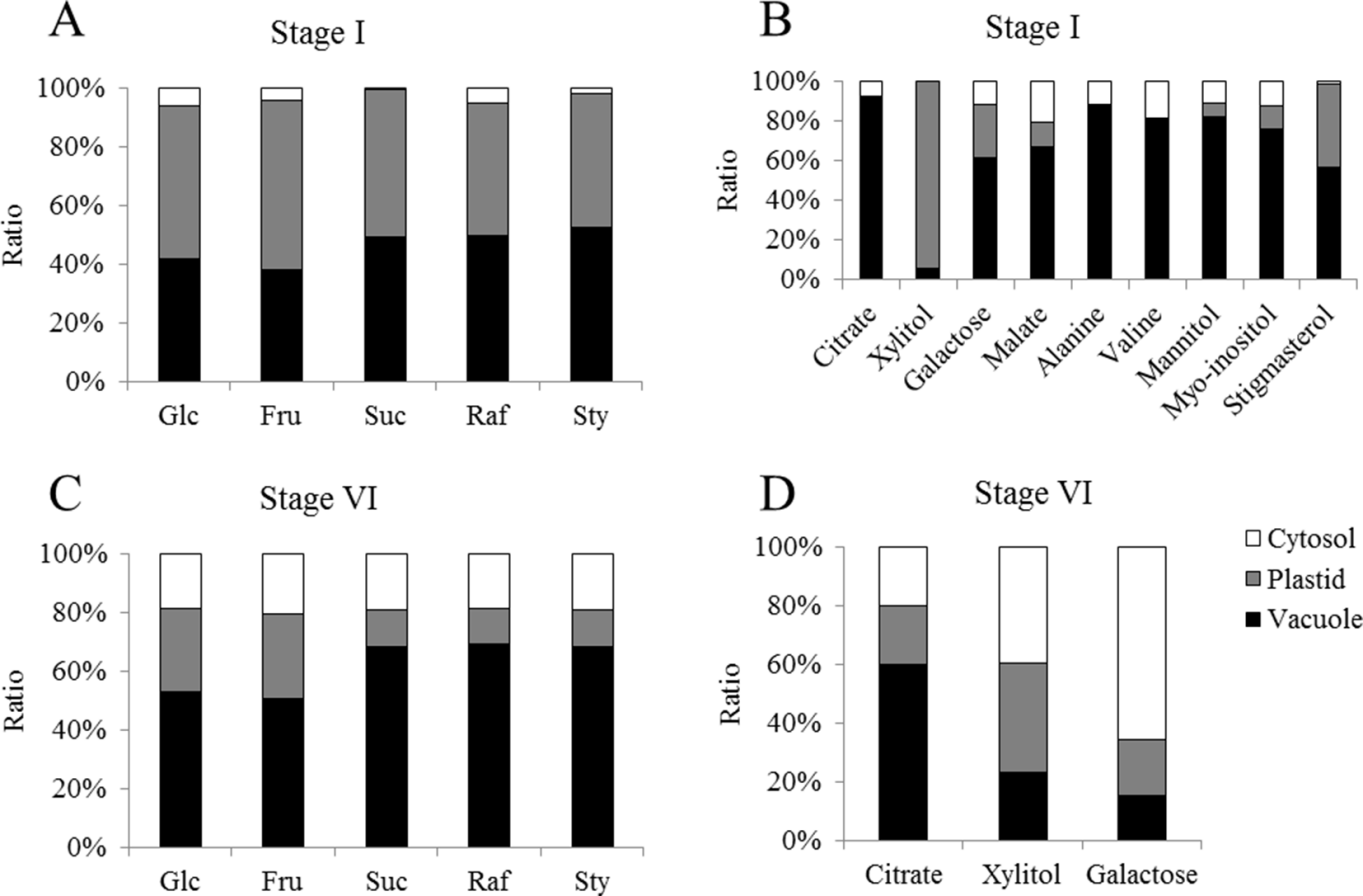
Relative distribution of metabolites over the cellular compartments cytosol (white), plastids (gray) and vacuole (black) from cotyledons of *Erythrina speciosa* seeds at two developmental stages (stage I, active metabolism; stage VI, dormant seeds). Values are given as mean of four biological replicates. Glc, glucose; Fru, fructose; Suc, sucrose; Raf, raffinose; Sty, stachyose. Relative abundance of sugars **(A)** and other metabolites **(B)** on stage I and sugars **(C)** and other metabolites **(D)** on stage VI.

The concentration of glucose, fructose, sucrose, and raffinose were evaluated by HPLC in the different fractions obtained from cotyledons from the two selected developmental stages. In general, we could observe that concentrations of glucose and fructose tend to be higher in stage I (**Figures 7A, B**). This pattern can also be observed for sucrose in the fractions enriched with cytosol. On the other hand, a greater concentration of raffinose is present in the later stages of development (**Figures 7A–D**).

## Discussion

As expected for seeds with orthodox behavior, the water content in *E. speciosa* seeds decreased during maturation until it reached ca. 10%, when germination reached 100%. It is important to emphasize that seed germination at stage VI without scarification was *ca.* 10% (data not shown) and that the highest values shown in Fig. 2B correspond to scarified seeds. Seed coat impermeability in *E speciosa* seeds is a frequent, but variable, occurrence. Both its occurrence and degree are dependent on climatic conditions during seed maturation (Molizane et al., 2018). Thus, in this work, the dormancy was near the maximum and was probably established in a very short period (stages V and VI), coinciding with the highest reduction in the water potential (**Figure 2A**), which corresponds to hydration level 1, according to Vertucci (1990).

The low content of starch, as previously reported by Mello et al. (2010), together with the high abundance of soluble sugars and the increased accumulation of RFO during seed maturation (**Table 2**) are also in agreement with the orthodox behavior of *E. speciosa*. Interestingly, the highest increase in raffinose accumulation in the embryonic axis and cotyledons of these seeds was observed from stage II to IV, before the sharp drop observed in the seed water content (**Figure 2**). In contrast, in *Inga vera*, a legume that has recalcitrant seeds, sucrose, and starch were the major reserves in mature seeds, and RFO were not detected all over the seed development and maturation (Caccere et al., 2013).

Investigation of seed cell walls of *E. speciosa* revealed a composition similar to that of other leguminous seeds, but with high levels of arabinose, as reported for marama beans (Mosele et al., 2011). Accumulation of arabinose in the cell walls was observed in the embryonic axis at the beginning of seed development of *E. speciosa*, before the highest decrease in water content. Conversely, a reduction in the proportion of this sugar from 40% to 35% was observed in the cotyledons from stage I to VI, respectively (**Table 1**). High proportion of arabinose, ranging from 38% to 60% of the cell walls, has been found in some orthodox seeds (Shiga and Lajolo, 2006; Gomez et al., 2009) contrasting with recalcitrant seeds as *I. vera*, in which the low proportion of arabinose in the cell walls was found in the embryonic axis (Caccere et al., 2013).

The changes observed in the seed cell wall composition of *E. speciosa* are probably due to cell wall adaptation to the maturation. High levels of arabinose, supposedly from long side chains of rhamnogalacturonan I, has been related to the increase of cell wall flexibility, by diminishing strong interactions among acidic pectic polysaccharides during tissue water loss (Moore et al., 2008). In leaf tissues of resurrection plants, the abundance of arabinose-containing polymers has been associated with the ability to survive the desiccation and rehydration process (Moore et al., 2009). Therefore, it is reasonable to suppose that the increased arabinose content in embryonic axis of *E. speciosa* has a role in the ability of cell walls to remain flexible during dehydration, avoiding cell wall damage due to water loss during seed maturation and contributing to its orthodox behavior. In *Arabidopsis* seeds, which do not contain starch, it was demonstrated that the arabinans accumulated in developing, and mature embryos are mobilized during seedling establishment (Gomez et al., 2009). Similar to *Arabidopsis*, *E. speciosa* has very little starch and accumulates arabinose-containing polymers during seed development. This suggests that arabinans, besides playing a role associated with desiccation tolerance during maturation of *E. speciosa* seeds, could also serve as a storage reserve, providing carbon to the embryo during early seedling growth.

Accompanying the water loss, there is a decay of metabolic activity in the seeds, which reduces the risk of ROS formation. Moreover, during maturation, seeds accumulate compounds, such as raffinose, which are supposed to protect the seeds against desiccation and ROS (Nishizawa et al., 2008). However, in *E. speciosa*, these biochemical changes (**Figure 3**) occurred early in seed maturation, when the water content was still high (**Figure 2A**), indicating a preparation of the metabolism for further water loss, which occurred primarily between stage IV and stage V (**Figure 2A**). This is even more evident when considering the water potential, which decreased from stage V on. According to Vertucci (1990), seeds from stage I to stage IV would be classified as hydration level 5, which means high level of hydration. Stages V and VI correspond to hydration levels 2 and 1, respectively.

Comparing different strategies of species propagation, *I. vera* seeds are dispersed with high content of water (hydration level), which allows storage only for a period shorter than 20 days at room temperature (Barbedo and Cicero, 2000). Caccere et al. (2013) studied these seeds observed high metabolic activity from the early stages of development until the end of the maturation, and Bonjovani and Barbedo (2019) showed that oxidative processes are involved in this activity. Our results indicate an opposite strategy in *E. speciosa* seeds, characterized by a dramatic decline in metabolism, which could classify them as orthodox. However, based on the hypothesis of Barbedo et al. (2013), seeds of *E. speciosa* simply complete their development, while this does not occur with *I. vera* seeds.

The changes observed in the embryonic axis can be explained by the need of reserve material from cotyledons. The decline of the metabolic level in axis of *E. speciosa* can also be explained by the decrease of sucrose, suggesting a reduction of substrate for glycolysis (**Figure 3**). The decay of metabolic activity can be inferred from the decrease in succinic, malic, and citric acids, which are intermediates of the Krebs cycle. Moreover, a general decrease in amino acids is also observed. Plants rarely oxidize amino acids for energy provision; however, amino acids can be used by other metabolic pathways at the central carbon metabolism and also provide carbon skeletons which may enter into the tricarboxylic acid cycle (TCA). Inside TCA cycle those can continue the respiration process or be used to produce other metabolites for different biosynthetic pathways (Nelson and Cox, 2001), such as synthesis of protective molecules involved in desiccation tolerance. Supporting this hypothesis, respiratory rates (**Figure 5**) decreased during maturation.

The accumulation of trehalose in the axes suggests an important role in desiccation tolerance, as previously observed in yeast and other organisms tolerant to desiccation (Crowe et al., 1992). This disaccharide, together with sucrose, may develop an important role in glass state formation (hydration level 1; Crowe et al., 1998) and might be involved as a protective molecule (de Klerk and Pumisutapon, 2008; Mollo et al., 2011), besides its function as a signal for sink activity (Smeekens et al., 2010).

According to Castillo et al. (1990), sucrose is the major soluble carbohydrate found in mature seeds of many species and acts as a substrate for metabolic reactions that occur at low temperatures. In a recent overview, Fàbregas and Fernie (2019 and references therein), reported experiments reveal that other sugars, such as RFOs, glucose, and fructose, accumulate earlier than other metabolites in response to stress. The authors emphasize a fine-tuned sequence of metabolic responses to desiccation with RFO accumulation followed by hexoses, sugar alcohols, and sucrose in leaf tissue of various species. The rapid accumulation of RFO, galactinol, and *myo*-inositol points to an important role during metabolic activity, which might include scavenging of ROS (Nishizawa et al., 2008). Rapid accumulation of raffinose was also obtained in *E. speciosa* seeds, where peaks for precursors *myo*-inositol, galactinol, and sucrose were observed already in stage II or III. Although raffinose likewise peaked in stage II, its decline in later stages was shallow as compared with sucrose, thus pointing to an important role also in mature seeds.

In axes, we observed a reduction in sucrose levels during maturation, suggesting that, in *E. speciosa*, this sugar seems not essential for the protection of cell membranes. Alternatively, sucrose may be used to synthesize RFOs (Castillo et al., 1990), which include raffinose, stachyose, and verbascose. Raffinose accumulation following stage II supports this hypothesis (**Table 1**). Thus, in *E. speciosa* seeds, sugars, such as trehalose and raffinose, appeared more directly involved in seed vitrification, which slows down enzymatic reactions. This event contributes to avoid degradation of cellular components of seeds and prevent membrane injuries and consequently the breakdown of cell compartments (Hoekstra et al., 2001), thus augmenting longevity of dry seeds (Leopold et al., 1994; Buitink et al., 2000). Also, as hypothesized by Walters (2015), the more stable the glasses, the longer the seed storability.

In studies carried out by Mello et al. (2010), raffinose and stachyose were the predominant sugars in embryonic axes and cotyledons of *E. speciosa* in addition to sucrose found in these seeds. According to Leprince et al. (1992) and Berjak and Pammenter (1999), the accumulation of RFO could be the result of a monosaccharide conversion, thus reducing the availability of respiration substrate, and consequently decreasing metabolic activity during desiccation and storage. This hypothesis may explain the high respiratory rates found at stage II, which decreased gradually until stage V (**Figure 5**), when physiological maturity was reached. In this stage of development, levels of amino acids and some organic acids were low (**Figure 3**), indicating a shift in carbon use (**Figure 4**) toward RFOs production as a means of decreasing the availability of respiration substrate. According to Mello et al. (2011), the precursor of raffinose synthesis, galactinol, could also be related to acquisition of desiccation tolerance in *Erythrina* seeds. Moreover, in a recent study, Hoermiller et al. (2017) inspected all metabolites quantitatively relevant in primary metabolism, i.e., amino-acids, carboxylic acids, amines, and sugar alcohols, and showed that cold acclimation causes substantial re-arrangements of metabolites within the cell. Sugars, especially those involved in raffinose metabolism, were shifted from the vacuole to the plastids (Hoermiller et al., 2017) to protect membranes involving the photosynthetic apparatus. The increased levels of galactinol during maturation observed in axes, together with decreased contents of pinitol and myo-inositol, which are substrates for the synthesis of galactinol, supports the idea that polyols may also be involved in acquisition of stress tolerance, i.e., desiccation, in seeds of *E. speciosa*, similar to the leguminous *Caesalpinia echinata* (Borges et al., 2006).

The analysis of subcellular distribution of metabolites revealed differences in the concentration of glucose, fructose, sucrose, and raffinose among subcellular compartments. The results showed an apparent increase of all sugars in the cytosol of mature cotyledons, when comparing stages I and VI (**Figure 7**). The concentrations of sugars in the vacuole also tended to increase. Similar results were previously observed by Knaupp et al. (2011), who compared non-acclimated and cold acclimated leaves of *Arabidopsis thaliana* and noted that sugars increased after acclimation in the cytosol. On the other hand, these authors found higher ratio of sucrose and raffinose in the plastids in the acclimated plants.

In *E. speciosa*, these sugars decreased during seed development and were not associated with plastids, especially in stage VI (**Figure 7**). Although RFO and related genes might be involved in cold (Bachmann et al., 1994; Egert et al., 2013) and freezing tolerance (Knaupp et al., 2011), as well as in desiccation tolerance (Steadman et al., 1996; Mello et al., 2011), their role as cellular protectants appears to be tissue and organism-dependent. While Knaupp et al. (2011) found a protective function in plastids, stabilizing the photosystem II, but not in the cytosol in *Arabidopsis* leaves, sucrose, raffinose, and stachyose became associated with the cytosol in seeds of *E. speciosa* during maturation. An additional role of oligosaccharides may reside in their ability to preserve the liquid crystalline state of cellular membranes in the dry state (Sun et al., 1994). Thus, a re-allocation of sucrose and RFO from vacuolar stores during seed maturation in *E. speciosa* might be an important prerequisite of seed longevity. Mobilization of these vacuolar stores might be responsible for the reduction of density that was observed when comparing distribution of subcellular compartments in density gradient for stage I and stage VI seeds (**Figure 6**).

**Figure 8:**
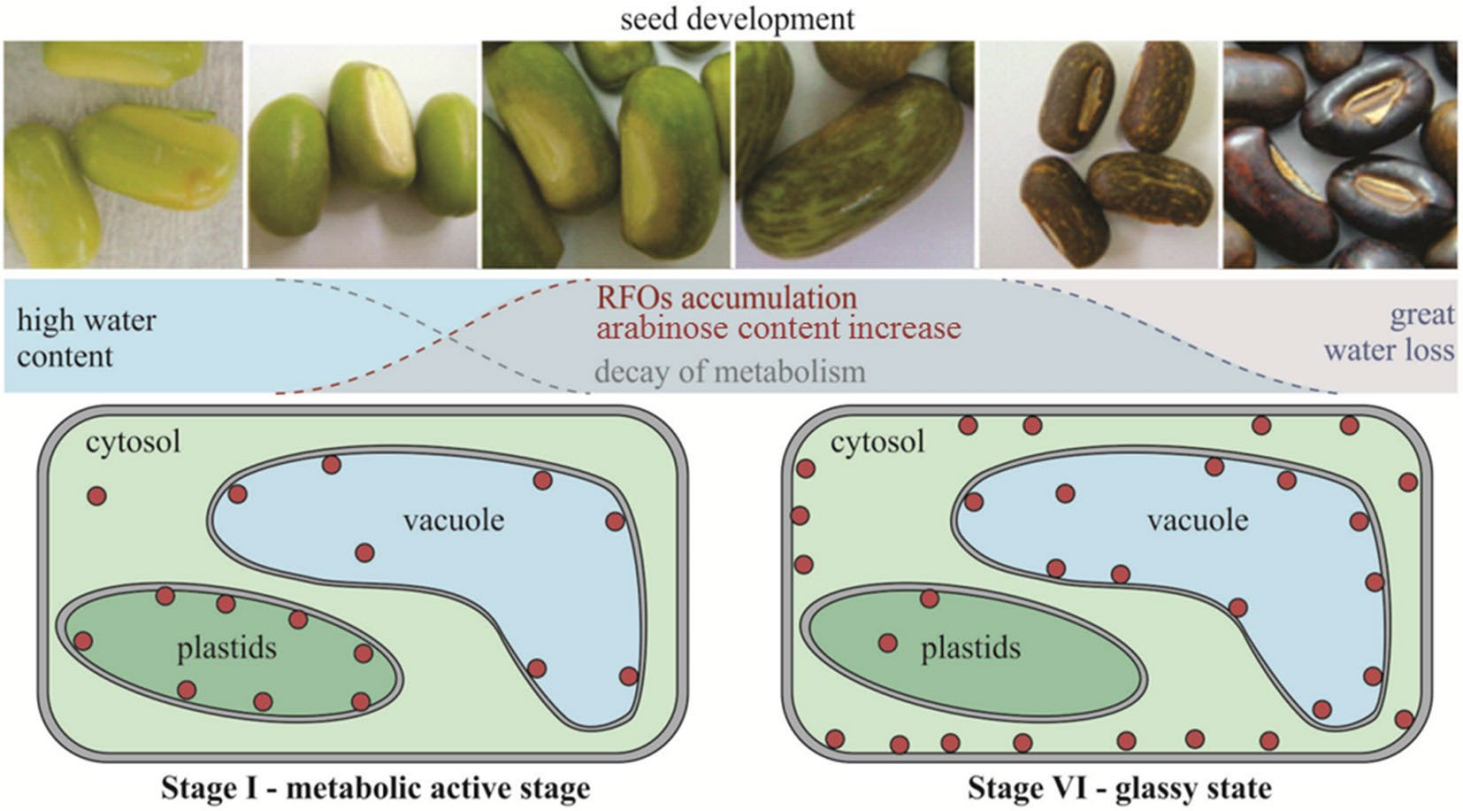
Schematic timeline of events of *Erythrina speciosa* seeds development. Red circles represent raffinose family oligosaccharides and their sub-cellular localization on cells of stage I and stage VI.

The combined results suggest, therefore, that in *E. speciosa* reduction of metabolic activity in seeds occurs mainly between the stages II and III before reaching the physiological maturity in the stage V of development. In addition, the accumulation of raffinose may also result from changing the use of carbon, decreasing the availability of substrate for respiration and therefore, the metabolic activity during the drying and storage, as illustrated in the diagram in **Figure 8**. This work confirms the hypothesis of the involvement of the RFO in the desiccation tolerance in seeds of *E. speciosa* and that its accumulation occurs in stages prior to major changes in water content. It highlights that the sub-cellular localization of RFO is crucial to protect cell membranes during seed development in the different cell compartments.

## Data Availability Statement

All datasets generated for this study are included in the manuscript/**Supplementary Files**.

## Author Contributions

AFH and FK obtained most of the experimental data. KS performed cell wall analysis. AGH and DC performed the non-aqueous fractionation analysis. AFH wrote the first draft of the manuscript. CB, MB, and DC designed the study. AFH, AGH, CB, MB, and DC contributed to the final version of the manuscript.

## Funding

This work was supported by FAPESP (2005/04139-7) and CNPq (478298/2011-0).

## Conflict of Interest

The authors declare that the research was conducted in the absence of any commercial or financial relationships that could be construed as a potential conflict of interest.

## Supplementary Material

Supplemental Figure 1 Morphological parameters of *Erythrina speciosa* seeds during development. Fruit length (A), Fruit diameter (B), Seed length (C), Seed width (D), Seed thickness (E) and Seed dry mass (F). Values are given as mean of six biological replicates considering fruit analysis (three fruits each replicate) and four biological replicates for seed analysis (ten seeds each replicate) ± Standard Deviation.

Supplemental Figure 2 Soluble carbohydrates profile by HPAEC/PAD from embryonic axes (left) and cotyledons (right) of Erythrina speciosa seeds during development. (a, d) 38, (b, e) 42 e (c, f) 44 days after anthesis. C - cyclitols; G - glucose; F - fructose; S - sucrose; R - raffinose; St - stachyose.

Supplemental Table 1 Correlation values between metabolites and marker enzymes (for sub-cellular compartments) and their respective *p* values at stage I and stage VI.
